# Differential rates of intravascular uptake and pain perception during lumbosacral epidural injection among adults using a 22-gauge needle versus 25-gauge needle: a randomized clinical trial

**DOI:** 10.1186/s12871-020-01137-0

**Published:** 2020-09-03

**Authors:** Robin Raju, Michael Mehnert, David Stolzenberg, Jeremy Simon, Theodore Conliffe, Jeffrey Gehret

**Affiliations:** 1 Department of Orthopedics and Rehabilitation, Yale New Haven Hospital/Yale University, 1 Long Wharf Drive, New Haven, CT 06511 USA; 2 Department of Physical Medicine and Rehabilitation, Rothman Orthopaedic Institute/Thomas Jefferson University Hospital, 925 Chestnut Street, Philadelphia, PA 19107 USA

**Keywords:** Intravascular uptake, Epidural, Transforaminal, Needle gauge, Fluoroscopy, Pain perception

## Abstract

**Background:**

Inadvertent intravascular injection has been suggested as the most probable mechanism behind serious neurological complications during transforaminal epidural steroid injections. Authors believe a smaller gauge needle may lead to less intravascular uptake and less pain. Theoretically, there is less chance for a smaller gauge needle to encounter a blood vessel during an injection compared to a larger gauge needle. Studies have also shown smaller gauge needle to cause less pain. The aim of the study was to quantify the difference between a 22-gauge needle and 25-gauge needle during lumbosacral transforaminal epidural steroid injection in regards to intravascular uptake and pain perception.

**Methods:**

This was a prospective single blind randomized clinical trial performed at outpatient spine practice locations of two academic institutions. One hundred sixty-two consecutive patients undergoing lumbosacral transforaminal epidural injections from February 2018 to June 2019 were recruited and randomized to each arm of the study – 84 patients were randomized to the 22-gauge needle arm and 78 patients to 25-gauge arm. Each transforaminal injection level was considered a separate incidence, hence total number of incidence was 249 (136 in 22-gauge arm and 113 in 25-gauge arm). The primary outcome measure was intravascular uptake during live fluoroscopy and/or blood aspiration. The secondary outcome measure was patient reported pain during the procedure on the numerical rating scale.

**Results:**

Fisher exact test was used to detect differences between 2 groups in regards to intravascular uptake and paired t-tests were used to detect differences in pain scores. The incidence of intravascular uptake for a 22-gauge needle was 5.9% (95% confidence interval: 1.9 to 9.8%) and for a 25-gauge needle, 7.1% (95% confidence interval: 2.4 to 11.8%) [*p* = 0.701]. Average numerical rating scale scores during the initial needle entry for 22-gauge and 25-gauge needle was 3.46 (95% confidence interval: 2.94 to 3.98) and 3.13 (95% confidence interval: 2.57 to 3.69) respectively [*p* = 0.375].

**Conclusions:**

The study showed no statistically significant difference in intravascular uptake or pain perception between a 22-gauge needle and 25-gauge needle during lumbosacral transforaminal epidural steroid injections.

**Trial registration:**

ClinicalTrials.gov NCT04350307. Registered 4/17/2020. (Retrospectively registered).

## Background

Over 40 million epidural injections are administered every year in the United States as per data obtained from Centers for Medicare & Medicaid Services [1]. Although generally considered a safe procedure, epidural steroid injections are not exempt from serious complications. There are three approaches to performing epidural injections – interlaminar approach, transforaminal approach and caudal approach. Transforaminal epidural steroid injections (TFESI) offer the advantage of placing steroids into ventral epidural space directly over the painful spinal nerve in patients with low back pain and/or leg pain. Hence many practitioners prefer this technique over interlaminar or caudal approach, although there is no definitive evidence to suggest one is superior to the other. Serious but rare complications have been reported with TFESI including spinal cord infarction, epidural hematoma, paralysis, and even death. Proposed mechanisms of action behind these devastating outcomes are arterial dissections/vasospasms, inadvertent intravascular injections or embolization of particulate corticosteroids [2–5].

Inadvertent intravascular injection has been suggested as the most probable mechanism behind serious neurological complications during TFESI [4–6]. The incidence of inadvertent intravascular injection during TFESI has been estimated to be 6–26% depending on the level of the injection [7–12]. A review of current literature reveals several studies evaluating factors involved in intravascular uptake during TFESI such as needle type, level of injection, injection approach, underlying comorbidities and so on. Among all these factors, needle type has been studied the most. Different bevel types do not appear to be a substantial factor in intravascular uptake during TFESI [13–17]. Although not conclusive, blunt type needles have shown a trend towards decreased intravascular uptake in few studies [14, 16]. In regards to level of injection, increased vascularity has been reported at the sacral foramen and at other spinal foramen (especially cervical, thoracic and higher lumbar levels) which can lead to increased intravascular uptake [11, 18, 19]. The artery of Adamkewicz, a major radicular artery often implicated in spinal cord infarction during TFESI, has been reported in anatomical studies to be found not only at higher lumbar levels but also at lower lumbar levels [20–22]. Several lumbosacral transforaminal epidural approaches targeting different parts of neuroforamen have been studied and no single approach has shown to be superior to the others in reducing intravascular uptake [17, 23, 24].

Although several needle types have been studied in the past, needle gauge has never been assessed with respect to intravascular uptake. It can be beneficial to know whether needle size plays a factor in intravascular uptake during TFESI. This study aims to look at two needle sizes – 22-gauge and 25-gauge needle. It can be hypothesized that 25-gauge needle due to its smaller diameter can potentially lead to less intravascular uptake. Theoretically, when taken into account the surface area covered by a needle while it traverses through tissue planes, there is less chance for a smaller diameter needle to encounter/puncture a blood vessel during an injection compared to a larger diameter needle. Studies have also shown smaller diameter needle to cause less pain [25]. Authors hypothesize that the smaller 25-gauge needle can be less painful for patients, hence making the procedure more tolerable. On the other hand, most practitioners tend to prefer 22-gauge needle for TFESI as it is easier to steer through tissue planes.

There are multiple ways to assess intravascular uptake during TFESI. Traditionally, blood aspiration, local anesthetic test dose and/or live fluoroscopy have been used to detect intravascular uptake during spinal procedures, but none of these methods have shown to be particularly sensitive. Digital subtraction angiography (DSA) has gained traction over the last decade and it has shown to be much more sensitive than other methods in detecting intravascular uptake [7, 26–28]. Although very sensitive, DSA has not been routinely used on lumbosacral TFESI due to the cost and increased radiation exposure to providers (and to patients) associated with its use.

## Methods

Institutional Review Board approval was obtained prior to the initiation of this study. There was no funding source involved in this study. Data is presented in accordance with the Consolidated Standards of Reporting Trials (CONSORT) statement and is available as Supplement. All patients provided written informed consent before participation in the study. Consecutive patients at two academic institutions from February 2018 to June 2019 were enrolled in the study. Injections were performed by five fellowship-trained interventional pain physicians. All injections were administered with Quincke needles (sharp bevel) and performed in outpatient fluoroscopy suites. Authors chose sharp bevel needle as it is the most commonly used needle in transforaminal epidural injections. 25-gauge blunt or short bevel needle is hard to steer through tissue planes lending itself limited clinical utility in transforaminal epidural injections.

Inclusion criteria included 1) patients with low back pain and/or radicular pain, 2) patients scheduled for lumbosacral TFESI. Exclusion criteria included 1) patients with contrast/local anesthetic allergy, 2) patients with pregnancy, coagulopathy, systemic infection, and inability to provide informed consent, 3) vulnerable patient population including prisoners, 4) patients with severe anxiety, 5) patients with prior lumbar surgery, 6) age < 18 years old, and 7) Body Mass Index (BMI) > 40.

**Fig. 1:**
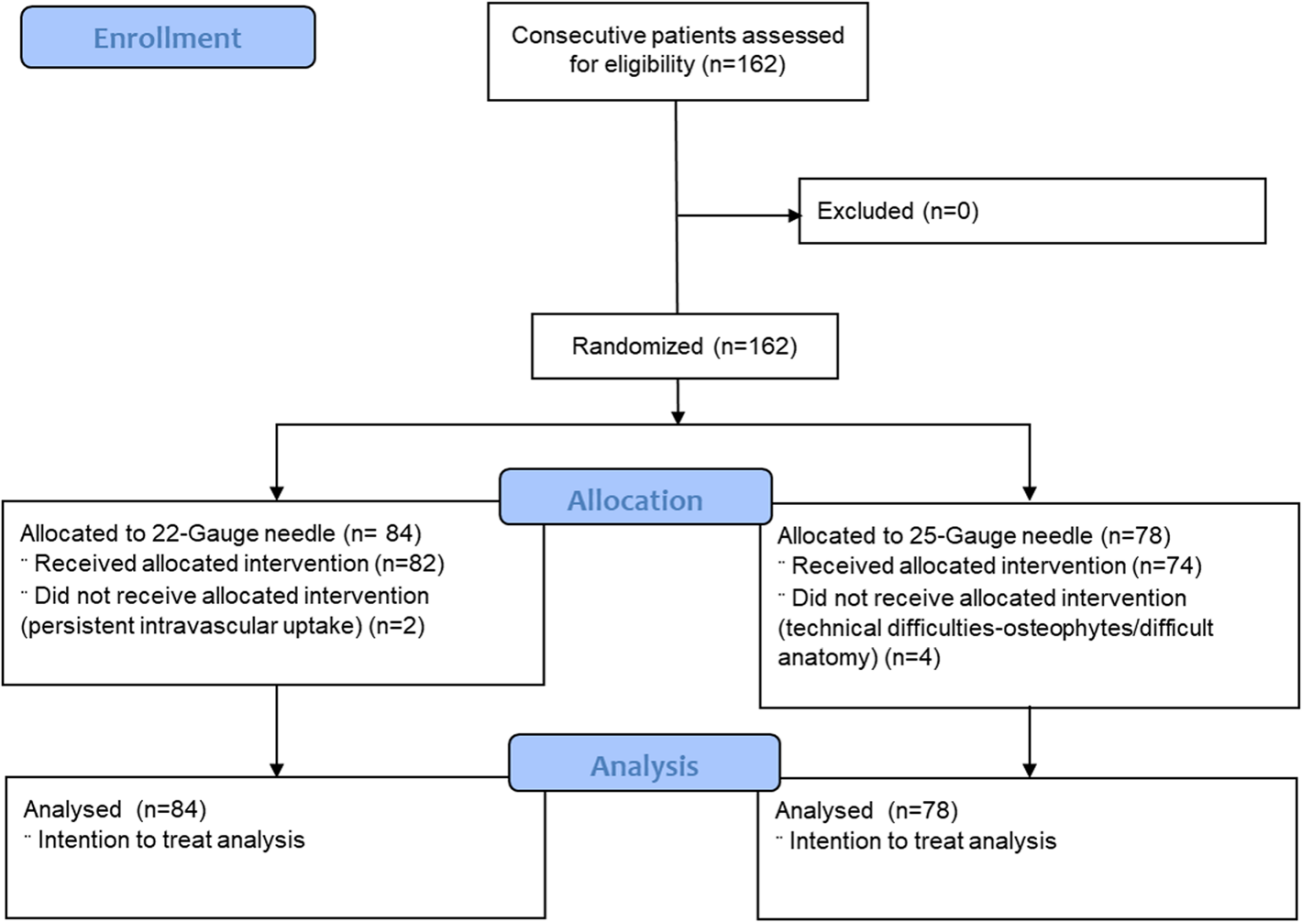
Study design

One hundred sixty-two consecutive patients were recruited and randomized to each arm of the study – 22-gauge vs 25-gauge (randomization was done separately for each provider based on a computer generated algorithm). Eighty-four patients were randomized to 22-gauge arm and 78 patients to 25-gauge arm (Fig. 1). Initial goal was to recruit around 250 patients, but due to logistical reasons and difficulty in recruitment, study was terminated early after enrolling 162 patients.

All injections were administered by Physical Medicine and Rehabilitation (physiatry) specialists who had completed a fellowship in interventional spine procedures and had at least 1 year of experience in interventional spine care. All practitioners used a similar approach for lumbosacral TFESI – subpedicular/supraneural approach. The target for L1-L5 TFESI was at the superior and posterior aspect (six o’clock position of the pedicle in the AP projection) of the lumbar neuroforamen. The target for S1 TFESI was at the superolateral aspect of dorsal S1 foramen. Providers were allowed to switch the TFESI approach to infraneural ‘Kambin’s’ triangle or interlaminar/caudal approach if the planned injection could not be administered with the initial approach.

The primary outcome measure was intravascular uptake during live fluoroscopy and/or blood aspiration. DSA was not available at all study locations and was therefore not utilized. The secondary outcome measure was patient reported pain during the procedure from 1 to 10 on the numerical rating scale (NRS).

After obtaining informed consent, patients were asked to record pre-procedure pain level on the NRS. Study coordinator explained the study design to the patients and specifically asked patients to pay attention to pain scores during 2 occasions – first one, during the initial needle entry after they feel the burn of the numbing agent and the second, during the administration of the steroids towards the end of the procedure. Interventionalists would also remind the patients to assess their pain during these 2 phases of the procedure. Patients were then brought to the fluoroscopy suite and placed in prone position on the fluoroscopy table. A procedural time-out was then conducted as per facility guidelines. The patients were prepped and draped in a standard sterile fashion in the prone position and the C-arm was positioned so that an oblique view of the neuroforamen was visualized and target marked. The soft tissues overlying this structure were infiltrated with 1–5 mL of 1% lidocaine (10 mg/mL) without epinephrine using a 27-gauge 1.5 in. needle. Lidocaine was injected at the skin (without creating a significant skin wheel) and through the subcutaneous tissue to 1 to 1.5 in. depth (maximum of 2 passes through the subcutaneous tissue). Then, a 22-gauge or 25-gauge (as per randomization) Quincke needle was inserted toward the target using an ipsilateral oblique trajectory view. Patients were blinded to the gauge of the needle. The needle was advanced under an oblique, AP and lateral visualization, to confirm correct needle tip placement. Aspiration was confirmed to be negative for cerebrospinal fluid and/or blood. Then a 1–2 mL volume of contrast dye (Omnipaque-240/Iohexol 240 mg/mL) was injected under live fluoroscopy to look for intravascular uptake. Needle tip was repositioned until an epidural only contrast pattern was observed prior to injecting the steroid. After obtaining satisfactory contrast flow pattern and negative blood aspiration, the injectate (3 mL of injectate per level - mixture of 1–2 mL of dexamethasone 10 mg/mL, 1-2 mL of 1% lidocaine and/or 1-2 mL of normal saline) was administered at each level along the nerve root and into the epidural space very slowly based on patient tolerance. Patients were reminded by interventionalists to assess their pain during this time. For the purposes of this study, only the initial contrast pattern was utilized. If an epidural only contrast flow was not obtained or persistent intravascular uptake was noted despite multiple needle redirections, the procedure was either abandoned or another approach was utilized (infraneural vs caudal vs interlaminar approach) as per patient and provider discretion. Live fluoroscopy and blood aspiration were utilized to confirm intravascular uptake at every injection level. Intravascular injection noted by either method was reported as ‘present’ for each level. Digital subtraction angiography or lidocaine test dose method was not used to confirm intravascular spread. At the conclusion of the procedure, all needle(s) were re-styletted, withdrawn and sterile dressings were placed. Patients were then brought to the recovery area and study coordinator or nurse (who had no knowledge of needle allocation) presented patients with written post-procedural questionnaire (paper form). Patients were specifically asked to rate the pain during the initial needle entry and also during the administration of injectate (steroid mixture). Overall tolerability of the procedure was also measured on an ordinal scale (‘well tolerated’ at 1 and ‘poorly tolerated’ at 4).

### Statistical analysis

Each needle entry at any given lumbosacral level was considered a separate incidence. For instance, a bilateral L5 TFESI was considered 2 separate incidences. Fisher exact test was used to detect differences between 2 groups in regards to intravascular uptake and paired t-tests were used to detect differences in pain scores. Both primary and secondary outcome measures were analyzed based on intent-to-treat principle.

## Results

A total of 249 TFESI injections were completed on 162 subjects. No serious complications were reported in any patients. Baseline demographics for both study groups are listed in Table 1.

**Table 1:** Patient baseline demographic data

	22-gauge (*n* = 84)	25-gauge (*n* = 78)	*p*-value
Age (years)	60.0	57.7	0.322
Sex (M:F)	M-45%; F-55%	M-52%; F-48%	0.543
Diagnosis			
-Radiculopathy	73.8%	80.7%	
-Spinal Stenosis	22.6%	14.1%	
-Other	3.6%	5.2%	

Eighty-seven patients received 2 level injections and the remaining 75 patients received 1 level injections. Each level was considered a separate incidence, hence total number of incidence was 249. Patients enrolled had TFESI at L2 to S1 levels. The most common level of injection was L5 neuroforamen for both groups. In four patients, the target could not be obtained (all in 25-gauge arm) due to technical difficulties (osteophytes/difficult anatomy) hence reassigned to 22-gauge needle arm and was completed successfully. Two other patients in 22-gauge group (both at S1 neuroforamen) were reassigned to interlaminar or caudal approach due to persistent intravascular uptake. All other patients had successful completion of the injection according to their assignments.

**Fig. 2:**
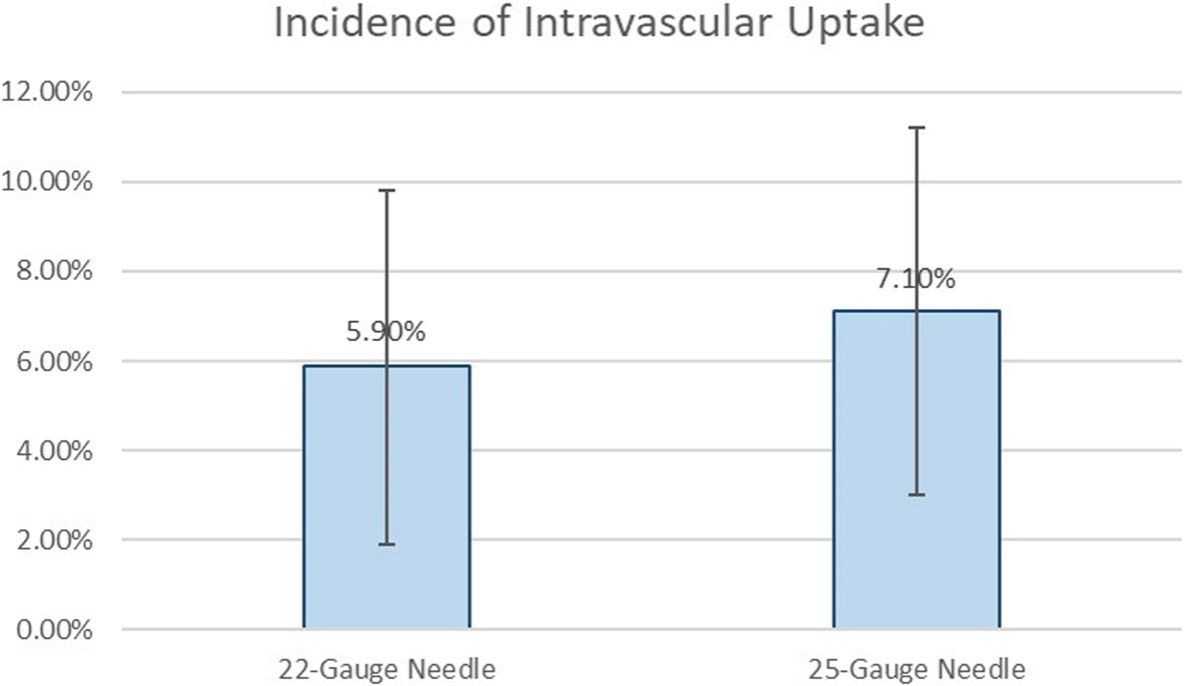
The incidence of intravascular uptake for 22-gauge group was 5.9% (8 out of 136) and 25-gauge group was 7.1% (8 out of 113) (*p* = 0.701)

The overall incidence of intravascular uptake for both the 22-gauge and 25-gauge group was 6.4% (16 out of 249) in this study. The incidence of intravascular uptake for 22-gauge group was 5.9% (8 out of 136, 95% confidence interval: 1.9 to 9.8%). The incidence of intravascular uptake for 25-gauge group was 7.1% (8 out of 113, 95% confidence interval: 2.4 to 11.8%). There was no statistically significant difference between both groups in regards to intravascular uptake (*p* = 0.701, Fig. 2).

Further analysis of intravascular uptake between different levels yielded no statistically significant differences (Table 2). There was a trend towards increased intravascular uptake incidence at S1 level among both groups, but was not statistically significant (*p* value = 0.767, Table 2).

**Table 2:** Intravascular uptake incidence per level

Levels	22-gauge (*n* = 136)	25-gauge (*n* = 113)	*p*-value
L2	1/4	0/7	
L3	0/17	3/19	
L4	0/33	0/32	
L5	2/66	0/32	
S1	5/16	5/23	0.767
Overall incidence	8/136 (5.9%)	8/113 (7.1%)	0.701

Pain scores (NRS) for 22-gauge and 25-gauge groups are listed in Table 3. There was no statistically significant difference in pain scores (during initial needle entry and administration of injectate) between both groups (Table 3). Injection tolerability was also measured (well tolerated =1, poorly tolerated = 4) and average score for 22-gauge group and 25-gauge group was 1.24 and 1.23 respectively (*p* = 1.000). Again, both groups showed no statistically significant difference in overall tolerability.

**Table 3:** Pain scores (NRS 1–10) between 22-gauge and 25-gauge groups

Pain scores	22-gauge (95% CI^a^) *n* = 84	25-gauge (95% CI^a^) *n* = 78	*p*-value
Average NRS before the procedure	5.88 (5.42 to 6.34)	6.21 (5.73 to 6.69)	0.330
Average NRS during the initial needle entry	3.46 (2.94 to 3.98)	3.13 (2.57 to 3.69)	0.375
Average NRS during the administration of steroid	4.01 (3.44 to 4.58)	3.77 (3.20 to 4.34)	0.554

There was no statistically significant difference in pain scores between 22-gauge and 25-gauge needle at various stages of the procedure

^a^*CI* confidence interval

## Discussion

The results of this study showed no statistically significant difference between the use of a 22-gauge and 25-gauge needle in regards to intravascular uptake or pain scores. One would expect the smaller gauge needle to reduce incidence of intravascular uptake but this study did not demonstrate any benefit in using a smaller bore 25-gauge needle as opposed to a larger bore 22-gauge needle. On the contrary, there seemed to be some disadvantage in using the 25-gauge needle as it can be hard to penetrate firm ligaments/skin and navigate around osteophytes, hence harder to steer and reach the target. Even though this study excluded patients with BMI > 40, all interventionalists found it less desirable to use 25-gauge needle as it took longer and required more fluoroscopic images to complete the procedure. In addition, it is to be noted that there were 6 cases in this study which switched assignment after randomization, and 4 of them were in 25-gauge arm (all of them due to technical challenge in obtaining the target at L4 and/or L5 levels). The other 2 assignment changes were due to persistent intravascular uptake, hence reassigned to interlaminar approach. Although not quantified in this study, 25-gauge needle can be difficult to steer through deeper tissue planes limiting its potential use in clinical practice. In this study, there was no outcome measure to gauge the difficulty level associated with completing a lumbosacral TFESI using 25-gauge needle. Authors recommend future studies looking at procedure time/exposure to further quantify this phenomenon in a more objective manner.

Although patients with lumbar surgery were excluded from the study, low thoracic and sacral surgeries can also result in increased vascularity in lumbar region due to post-surgical changes. On post-hoc analysis, none of the patients had documented low thoracic or sacral surgeries prior to enrolling in the study.

The study may also suggest that a smaller gauge needle may not lead to less pain. Although the study was not well designed to detect the differences in pain perception, the results suggest no difference in pain scores or tolerability between the 2 groups. Patients were specifically asked to rate the pain during the initial needle entry and also during the administration of injectate (steroid mixture) hoping to differentiate pain experienced at various stages of the procedure, but no statistically significant differences were observed.

### Limitations

Similar to prior studies on intravascular uptake, one of the limitations of this study was also the relatively small sample size. A sample size of 249 was not enough to detect small changes that may exist between the 2 groups especially given the low overall incidence of intravascular uptake during TFESI. Also true randomization at injection event level was not possible as one patient could have had multiple events. Although initial goal was to recruit around 250 patients, study was terminated early at 162 patients, hence an ‘event’ was changed from patients to level of injections for statistical analysis possibly implying cluster randomization.

Authors did not use digital subtraction angiography to detect intravascular uptake as it was not available at all study locations. Instead, standard live fluoroscopy and/or vascular aspiration were used. Studies have shown DSA to increase the sensitivity of detecting intravascular uptake [7, 26–28].

Pain scores were obtained after the procedure when patients were in the recovery room which could have led to recall bias. When patients were asked to rate pain during the initial needle entry, they could in fact be assessing the needle used for local anesthetic infiltration. More accurate pain scores could have been obtained if asked during the procedure immediately after placement of the needle and administration of steroids. Although the procedure was standardized in the research protocol, there may still be differences in how the injection was performed between various interventionalists (five in this study) which can affect patients’ pain perception. Sensitivity analysis (taking into consideration individual provider variability) appeared to affect pain scores but had limited impact on the rate of intravascular uptake.

## Conclusions

One would expect the smaller 25-gauge needle to reduce incidence of intravascular uptake and be less painful during TFESI, but the study finds no conclusive evidence. In conclusion, the study showed no statistically significant difference in intravascular uptake or pain perception between a 22-gauge needle and 25-gauge needle during lumbosacral TFESI.

## Supplementary information

**Additional file 1.**

## Data Availability

All data is available from the corresponding author upon request.

## Abbreviations

TFESI: Transforaminal epidural steroid injection
BMI: Body Mass Index (BMI)
DSA: Digital subtraction angiography (DSA)
NRS: Numerical rating scale
