# Supplementary material for: Differential rates of intravascular uptake and pain perception during lumbosacral epidural injection among adults using a 22-gauge needle versus 25-gauge needle: a randomized clinical trial
Source: BMC Anesthesiol. 2020 Sep 3;20:222. doi: 10.1186/s12871-020-01137-0 (PMC7469311; doi:10.1186/s12871-020-01137-0)
Supplement: Supplementary file 1 — Additional file 1. [file 12871_2020_1137_MOESM1_ESM.docx]

**CONSORT 2010 checklist**

I. TITLE & ABSTRACT

Title:

Differential rates of intravascular uptake and pain perception during lumbosacral transforaminal epidural steroid injection using a 22-gauge needle versus 25-gauge needle

Abstract

Background: Inadvertent intravascular injection has been suggested as the most probable mechanism behind serious neurological complications during transforaminal epidural steroid injections (TFESI).

Objectives: To quantify the difference between a 22-gauge needle and 25-gauge needle during lumbosacral transforaminal epidural steroid injection in regards to intravascular uptake and pain perception.

Study Design: Prospective single blind randomized clinical trial.

Setting: Outpatient spine practice at two academic institutions.

Methods: 162 consecutive patients undergoing lumbosacral transforaminal epidural injections were recruited and randomized to each arm of the study – 84 patients were randomized to the 22-gauge needle arm and 78 patients to 25-gauge arm. A total of 249 transforaminal epidural steroid injections were completed on 162 subjects. The primary outcome measure was intravascular uptake during live fluoroscopy and/or blood aspiration. The secondary outcome measure was patient reported pain during the procedure on the numerical rating scale.

Results: The incidence of intravascular uptake for a 22-gauge needle was 5.9% and for a 25-gauge needle, 7.1% (p=0.701). Average numerical rating scale scores during the initial needle entry for 22-gauge and 25-gauge needle was 3.46 and 3.13 respectively (p=0.375).

Limitations: A sample size of 249 was not enough to detect small changes that may exist between the 2 groups especially given the low overall incidence of intravascular uptake during transforaminal epidural steroid injections. Authors did not use digital subtraction angiography to detect intravascular uptake.

Conclusions: The study showed no statistically significant difference in intravascular uptake or pain perception between a 22-gauge needle and 25-gauge needle during lumbosacral transforaminal epidural steroid injections.

II. INTRODUCTION

Background:

Inadvertent intravascular injection has been suggested as the most probable mechanism behind serious neurological complications during TFESI. Although several needle types have been studied in the past, needle gauge has never been assessed with respect to intravascular uptake. There is also a notion that smaller gauge needle tends to be less painful for patients, hence making the procedure more tolerable.

Objective:

To quantify the difference between a 22-gauge needle and 25-gauge needle during lumbosacral transforaminal epidural steroid injection in regards to intravascular uptake and pain perception.

III. METHODS

a. Trial design

Prospective single blind randomized clinical trial. 162 consecutive patients undergoing lumbosacral transforaminal epidural injections were recruited and randomized to one of the two arms of the study. Randomization was done separately for each provider based on a computer generated algorithm.

B. Participants

Consecutive patients at two academic institutions from February 2018 to June 2019 were enrolled for study. All injections were administered with Quincke needles (sharp bevel) and performed in outpatient fluoroscopy suites.

Inclusion criteria included 1) patients with low back pain and/or radicular pain, 2) patients scheduled for lumbosacral TFESI. Exclusion criteria included 1) patients with contrast/local anesthetic allergy, 2) patients with pregnancy, coagulopathy, systemic infection, and inability to provide informed consent, 3) vulnerable patient population including prisoners, 4) patients with severe anxiety, 5) patients with prior lumbar surgery, 6) age <18 years old, and 7) Body Mass Index (BMI) > 40

C. Interventions

All patients underwent transforaminal epidural injection. Procedure was performed with either 22-gauge vs 25-gauge needle based on randomization.

D. Outcomes

The primary outcome measure was intravascular uptake during live fluoroscopy and/or blood aspiration. The secondary outcome measure was patient reported pain during the procedure from 1-10 on the numerical rating scale (NRS).

E. Sample size

A total of 249 TFESI injections were completed on 162 subjects. 87 patients received 2 level injections and the remaining 75 patients received 1 level injections. Each level was considered a separate incidence, hence total number of incidence was 249.

F. Randomization – sequence generation

Randomization was done separately for each provider based on a computer generated algorithm. There were 5 interventionalists involved in the study. Interventionalists would only know the assigned needle just prior to the injection.

I. Blinding (masking)

Single blinded study – Patients were blinded to the gauge of the needle. Interventionalists would only know the assigned needle just prior to the injection.

J. Statistical methods

Each needle entry at any given lumbosacral level was considered a separate incidence. For instance, a bilateral L5 TFESI was considered 2 separate incidences. Fisher exact test was used to detect differences between 2 groups in regards to intravascular uptake and t-tests were used to detect differences in pain scores. Both primary and secondary outcome measures were analyzed based on intent-to-treat principle.

IV. RESULTS

1. Participant flow

Consecutive patients at two academic institutions from February 2018 to June 2019 were enrolled for study. All injections were performed in outpatient fluoroscopy suites. After obtaining informed consent, patients were asked to record pre-procedure pain level on the NRS. Patients were then brought to the fluoroscopy suite and placed in prone position on the fluoroscopy table for the procedure. A procedural time-out was then conducted as per facility guidelines. At the conclusion of the procedure, all needle(s) were re-styletted, withdrawn and sterile dressings were placed. Patients were then brought to the recovery area and presented with the post-procedural questionnaire.

1. Recruitment

Consecutive patients scheduled for transforaminal epidural injections at two academic institutions from February 2018 to June 2019 were approached by study coordinator/physician and informed of the study. If interested, patients would be enrolled in the study.

C. Baseline data

Table 1: Patient baseline demographic data

|  | 22-gauge (n=84) | 25-gauge (n=78) | p-value |
| --- | --- | --- | --- |
| Age (years) | 60.0 | 57.7 | 0.322 |
| Sex (M:F) | M-45%; F-55% | M-52%; F-48% | 0.543 |
| Diagnosis |  |  |  |
| -Radiculopathy | 73.8% | 80.7% |  |
| -Spinal Stenosis | 22.6% | 14.1% |  |
| -Other | 3.6% | 5.2% |  |

D. Numbers analyzed

Both primary and secondary outcome measures were analyzed based on intent-to-treat principle.

A total of 249 TFESI injections were completed on 162 subjects. 87 patients received 2 level injections and the remaining 75 patients received 1 level injections. Each level was considered a separate incidence, hence total number of incidence was 249.

84 patients received 22-gauge needle and 78 patients received 25-gauge needle.

Primary outcome:

The overall incidence of intravascular uptake for both the 22-gauge and 25-gauge group was 6.4% (16 out of 249) in this study. The incidence of intravascular uptake for 22-gauge group was 5.9% (8 out of 136, 95% confidence interval: 1.9% to 9.8%). The incidence of intravascular uptake for 25-gauge group was 7.1% (8 out of 113, 95% confidence interval: 2.4% to 11.8%). There was no statistically significant difference between both groups in regards to intravascular uptake (p=0.701).

Secondary outcome:

Pain scores (NRS 1-10) between 22-gauge and 25-gauge groups

| Pain scores | 22-gauge (95% CI*)  n = 84 | 25-gauge (95% CI*)  n = 78 | p-value |
| --- | --- | --- | --- |
| Average NRS before the procedure | 5.88 (5.42 to 6.34) | 6.21 (5.73 to 6.69) | 0.330 |
| Average NRS during the initial needle entry | 3.46 (2.94 to 3.98) | 3.13 (2.57 to 3.69) | 0.375 |
| Average NRS during the administration of steroid | 4.01 (3.44 to 4.58) | 3.77 (3.20 to 4.34) | 0.554 |

There was no statistically significant difference in pain scores between 22-gauge and 25-gauge needle at various stages of the procedure. *CI = confidence interval

E. Outcomes and estimation

Confidence intervals for both primary and secondary outcomes stated above.

F. Ancillary analyses

Intravascular uptake incidence also calculated per level

| Levels | 22-gauge (n=136) | 25-gauge (n=113) | p-value |
| --- | --- | --- | --- |
| L2 | 1/4 | 0/7 |  |
| L3 | 0/17 | 3/19 |  |
| L4 | 0/33 | 0/32 |  |
| L5 | 2/66 | 0/32 |  |
| S1 | 5/16 | 5/23 | 0.767 |
| Overall incidence | 8/136 (5.9%) | 8/113 (7.1%) | 0.701 |

There was a trend towards significance in regards to increased intravascular uptake incidence at S1 level among both groups (p value = 0.767).

G. Harms

No serious complications were reported in any patients.

V. DISCUSSION

A. Limitations

Similar to prior studies on intravascular uptake, one of the limitations of this study was also the relatively small sample size. A sample size of 249 was not enough to detect small changes that may exist between the 2 groups especially given the low overall incidence of intravascular uptake during TFESI. Ideally to detect a difference, a randomized clinical trial will require a sample size of greater than 1000 subjects.

Authors did not use digital subtraction angiography to detect intravascular uptake as it was not available in all study locations. Instead, standard live fluoroscopy and/or vascular aspiration were used. Studies have shown DSA to increase the sensitivity of detecting intravascular uptake (7,25,26,27).

Pain scores were obtained after the procedure when patients were in the recovery room which could have led to recall bias. More accurate pain scores could have been obtained if asked during the procedure immediately after placement of the needle and administration of steroids. Although the procedure was standardized in the research protocol, there may still be subtle differences in how the injection was performed between various interventionalists (five in this study) which can affect patients’ pain perception.

B. Generalizability

Increased generalizability can be inferred as the study protocol only had very few exclusion criteria.

C. Interpretation

A sample size of 249 was not enough to detect small changes that may exist between the 2 groups especially given the low overall incidence of intravascular uptake during TFESI. But 22 gauge doesn’t seem to dramatically increase the intravascular uptake during TFESI, hence very safe to use.

VI. OTHER INFORMATION

A. Registration

Institutional Review Board approval was obtained prior to the initiation of this study at both study locations. IRB study registration number at Rothman Orthopedic Institute was 17D.555. IRB study registration number at Yale University was 2000023982. The study is registered with ClinicalTrials.gov (NCT04350307).

B. Protocol

As above

C. Funding

None
